# Editorial: The role of nutrition in pediatric chronic diseases: a focus on metabolic, genetic, and palliative care challenges

**DOI:** 10.3389/fnut.2025.1747647

**Published:** 2026-01-08

**Authors:** Fiorentina Guida, Daniele Zama, Marcello Lanari

**Affiliations:** 1 Pediatric Unit, IRCCS Azienda Ospedaliero-Universitaria di Bologna, Bologna, Italy; 2 Department of Medical and Surgical Sciences, Alma Mater Studiorum, University of Bologna, Bologna, Italy

**Keywords:** pediatric chronic disease, inherited metabolic disease, asthma, pneumonia, malnutrition, inflammation, childhood cancer survivors

Nutritional management in children with chronic diseases represents a multifaceted challenge. Metabolic and endocrinological disorders often require individualized dietary approaches to ensure adequate growth and metabolic balance. Moreover, nutritional status plays a key role in the pathogenesis of several inflammatory pediatric conditions and can influence both the prevalence and severity of infectious diseases. Therapeutic interventions may further complicate nutritional management by inducing adverse effects that negatively affect nutritional status, whereas certain pharmacological treatments may, conversely, confer beneficial effects on cardiometabolic health.

This Research Topic investigates how nutritional status and dietary habits influence the development, progression, and management of specific congenital and acquired pediatric chronic diseases.

Inherited metabolic diseases (IMDs) require careful modulation of macronutrient intake and supplementation to prevent complications, optimize growth, and maintain metabolic homeostasis (1). Newborns and toddlers with IMDs need adequate protein intake to support anabolism, while avoiding excessive protein restriction that could lead to metabolic decompensation. Balancing breast milk volume with tolerance to specific amino acids and adjusting specialized infant formulas accordingly can be challenging. A wide variety of specialized infant protein substitutes (IPSs) exists to meet the diverse nutritional needs of infants with IMDs. Di Costanzo et al. analyzed and compared IPSs available in the Italian market, highlighting differences in macronutrient and micronutrient composition for Tyrosinemia type 1, Maple Syrup Urine Disease, Glutaric Aciduria type 1, Classical Homocystinuria, classic organic acidurias, and urea cycle disorders.

Emerging evidence indicates that dietary patterns and specific nutrients may have pro- or anti-inflammatory effects, modulating chronic disease risk and severity through influences on gut microbiota, oxidative stress, and immune function (2, 3). In asthma, persistent low-grade inflammation is a central pathogenic factor that can be influenced by diet.

Xu et al. explored the association between the Children's Dietary Inflammatory Index (C-DII) and asthma prevalence in U.S. children aged 6–19 years. Moderate C-DII scores were associated with the lowest asthma risk, while both higher and lower scores correlated with increased prevalence, suggesting that overly pro- or anti-inflammatory diets may disrupt immune homeostasis. Balanced, age-appropriate diets, combining anti-inflammatory and nutrient-dense foods, while moderating pro-inflammatory components, may support immune function, growth, and respiratory health. Younger children appeared particularly sensitive to dietary inflammatory factors, highlighting nutrition's critical role in early immune development.

Nutritional status also influences susceptibility to infectious diseases and treatment response. Undernutrition is a major risk factor for severe disease in children under five, increasing the likelihood of pneumonia, mortality, recurrent infections, and repeated hospitalizations (4, 5). Pneumonia further exacerbates undernutrition by increasing the metabolic demands, and children recovering from pneumonia often require increased energy intake. Ready-to-use supplementary food (RUSF) and ready-to-use therapeutic food (RUTF) are effective strategies for addressing moderate acute malnutrition in children. However, no interventions are specifically tailored for children with pneumonia. Nalwanga et al. conducted a multicenter case-control study in Uganda, investigating daily RUTF supplementation in undernourished children aged 6–59 months with severe pneumonia. Supplementing did not reduce adverse outcomes, suggesting that nutritional needs in severe pneumonia are not fully met by RUTF alone and emphasizing the importance of primary prevention of undernutrition.

Childhood and adolescent cancer survivors (CAcs) face a high risk for undernutrition during cancer treatment (6). They also have an increased risk of cardiovascular diseases secondary to cancer therapies, which may manifest decades after treatment, underscoring the need to prevent cardiometabolic risk factors. Promoting healthy lifestyles and physical activity is central to prevention (7, 8).

In children with acute lymphoblastic leukemia, undernutrition is associated with higher infection rates, treatment interruptions, and reduced survival, while adequate nutrition improves treatment tolerance and long-term outcomes (9). Providing practical guidance on drug–diet compatibility to CAcs and their families is challenging but essential for adherence to appropriate dietary practices (10). Zibaldo et al. evaluated the “Hematognam” app, a digital tool designed to support CAcs and their families in managing diet and healthy lifestyles. The app allows dual access for parents and children, depending on age and family preference, and features user data input, chemotherapy phase selection, and tailored dietary guidance, including recommended and discouraged foods, hygiene rules, and drug–food interactions. High usability scores indicated strong family acceptance, and prompt operator support enhanced its effectiveness, demonstrating the potential of digital tools as alternatives to telemedicine.

Similarly, Alhajri investigated the lifestyle habits of pediatric leukemia patients during treatment through a structured questionnaire covering demographics, physical activity, dietary habits, and factors influencing adherence to a healthy lifestyle. Caregivers and participants reported a positive relationship between physical activity, appetite, and overall health. The study revealed variations in lifestyle behaviors based on gender, age, leukemia type, and treatment stage. Males displayed higher diet quality, and children aged 8–9 years reported greater motivation for physical activity. Nevertheless, 60% of participants failed to meet recommended activity levels, with those in remission being less active than peers in earlier treatment phases, highlighting the need for tailored interventions according to age and treatment stage.

In contrast, specific anti-seizure medications (ASMs) appear to improve cardiometabolic parameters in children with epilepsy. Children with epilepsy often exhibit reduced high-density lipoprotein cholesterol (HDL-C) compared to healthy peers, a predictor of atherosclerosis (11).

Guo et al. demonstrated that children treated with enzyme-inducing ASMs, particularly oxcarbazepine, had higher HDL-C levels at diagnosis compared to untreated patients, although levels remained below those of healthy children. ASM therapy may contribute to reducing cardiovascular risk in this population.

In summary, the presented studies provide relevant data on the impact of nutritional status, dietary habits, and lifestyle interventions in pediatric chronic diseases. These papers highlight numerous aspects that require further clarification, including optimal dietary strategies for IMDs, the interplay between diet and inflammation, the management of nutrition during cancer treatment, and the long-term cardiometabolic effects of pharmacological therapies. After reviewing these findings, concepts such as individualized formulas for IMDs, age- and treatment-specific interventions for CAcs, and the use of innovative strategies to support nutrition and health will appear clearer to the reader, reinforcing the importance of tailored approaches to optimize growth and long-term wellbeing in children with chronic diseases.
